# Revision Neck Lift Surgery: A Systematic Review of Indications, Techniques, and Outcomes

**DOI:** 10.1007/s00266-026-05686-6

**Published:** 2026-02-11

**Authors:** Serhat Şibar, Ayhan Işık Erdal, Giray Genç

**Affiliations:** 1 Faculty of Medicine, Department of Plastic, Reconstructive and Aesthetic Surgery, Gazi University, 06500 Ankara, Turkey; 2 Private Practice, Istanbul, Turkey

**Keywords:** Revision neck lift, Platysmaplasty, Submandibular gland, Secondary rhytidectomy, Complications, Systematic review

## Abstract

**Background:**

Revision neck lift is among the most technically demanding facial rejuvenation procedures and often addresses complex deformities related to aging, prior operations, or inadequate management of deep neck structures. Evidence remains sparse and heterogeneous.

**Methods:**

A systematic review was performed in accordance with PRISMA 2020 and prospectively registered in PROSPERO (CRD420251110148). PubMed, Scopus, and Web of Science were searched without time restrictions. Studies reporting ≥5 patients who underwent revision neck lift were included. Data on demographics, indications, techniques, follow-up, and complications were extracted, and owing to heterogeneity and small sample sizes, results were synthesized descriptively.

**Results:**

Five retrospective series comprising 188 revision neck lift cases met the inclusion criteria. Among patients with reported sex, 95% were women, with reported ages between 31 and 77 years. Recurrent platysmal banding, persistent submental fullness, and submandibular gland (SMG) ptosis were the most frequently cited indications. Platysmal maneuvers were the most commonly reported revision interventions, although technique subtypes and denominators were inconsistently reported. SMG reduction was performed in 13% of patients and detailed in only one study, limiting any inference regarding indications, efficacy, or risk. Reported complications were uncommon and mainly transient, consisting primarily of transient marginal mandibular neuropraxia and isolated hematoma; no permanent nerve deficits were noted. Given heterogeneity and inconsistent reporting of complications across studies, complications were summarized descriptively without pooling.

**Conclusion:**

Available case series suggest potential contour improvement after revision neck lift; however, aesthetic outcomes were predominantly qualitative and surgeon-reported, and standardized objective or patient-reported measures were rarely used.

**Level of Evidence IV:**

This journal requires that authors assign a level of evidence to each article. For a full description of these Evidence-Based Medicine ratings, please refer to the Table of Contents or the online Instructions to Authors www.springer.com/00266.

## Introduction

Neck lift surgery is one of the most complex components of facial rejuvenation, and revision cases span a wide spectrum of technical challenges. Increasing demand for facial surgery at younger ages and an aging population have contributed to a rise in secondary and tertiary procedures [1–7]. Although the techniques, complication rates, and long-term outcomes of primary neck lift have been well described, data on revision surgery remain sparse and heterogeneous [2, 4, 7–10].

Revision may be required for iatrogenic deformities, the natural progression of aging, or inadequate management of the neck at the index operation [4, 5, 7–9, 11]. Common problems include recurrent platysmal bands, submental lipodystrophy, submandibular gland (SMG) ptosis, contour irregularities, and the “cobra-neck” deformity [5, 7, 9].

Compared with primary procedures, revision surgery is hampered by scar formation, distortion of dissection planes, altered facial nerve anatomy, loss of tissue elasticity, thinning of the SMAS layer, and new comorbidities [5, 6, 12, 13]. The widespread use of energy-based devices, thread lifting, and filler applications has added further complexity [13, 14].

Subplatysmal structures appear to be a more frequent source of recurrent problems than the supraplatysmal layer; over time, ptosis of the digastric muscles and SMG can produce contour irregularities, and tightening of the SMAS–platysma complex is often required to restore cervicomental definition [1, 2, 5].

The current literature consists mainly of small retrospective case series with nonstandardized outcome reporting and provides limited guidance for evidence-based decision-making. Therefore, a systematic evaluation of the indications, techniques, complication profiles, and aesthetic outcomes of revision neck lift surgery is warranted. This study synthesizes existing evidence to clarify when revision neck lift should be considered, which techniques are preferred, and what results and risks can realistically be expected.

## Materials and Methods

This systematic review was conducted in accordance with the PRISMA 2020 guidelines and was prospectively registered in PROSPERO (CRD420251110148) [15].

### Scope and Inclusion Criteria

Patients who had previously undergone neck lift or face–neck lift and later required revision neck lift surgery were eligible. Studies had to report original clinical data on more than five patients and be published in English. Exclusion criteria were series limited to primary cases, case reports with fewer than five revision patients, cadaveric or experimental studies, animal studies, non-English publications, and studies limited to simple scar revision or isolated skin-only procedures without true revision neck lift.

### Interventions

All surgical techniques used in revision neck lift were analyzed, including submental (corset) platysmaplasty, lateral platysmal suspension, SMAS or deep-plane modifications, SMG excision or suspension, subplatysmal fat excision, and skin redraping or excision. Minimally invasive procedures (energy-based devices, thread lifting, fillers, injection treatments) were not evaluated when performed alone but were recorded when used as adjuncts to surgery.

### Literature Search

Searches in PubMed, Scopus, and the Web of Science Core Collection were performed using combinations of the terms “revision neck lift,” “secondary neck lift,” “secondary rhytidectomy,” “facelift revision,” “platysmaplasty,” “submandibular gland,” “cervicoplasty,” and “neck rejuvenation.” No date limits were applied. Reference lists of included articles and of articles citing them were manually screened for additional studies.

### Study Selection

**Fig. 1:**
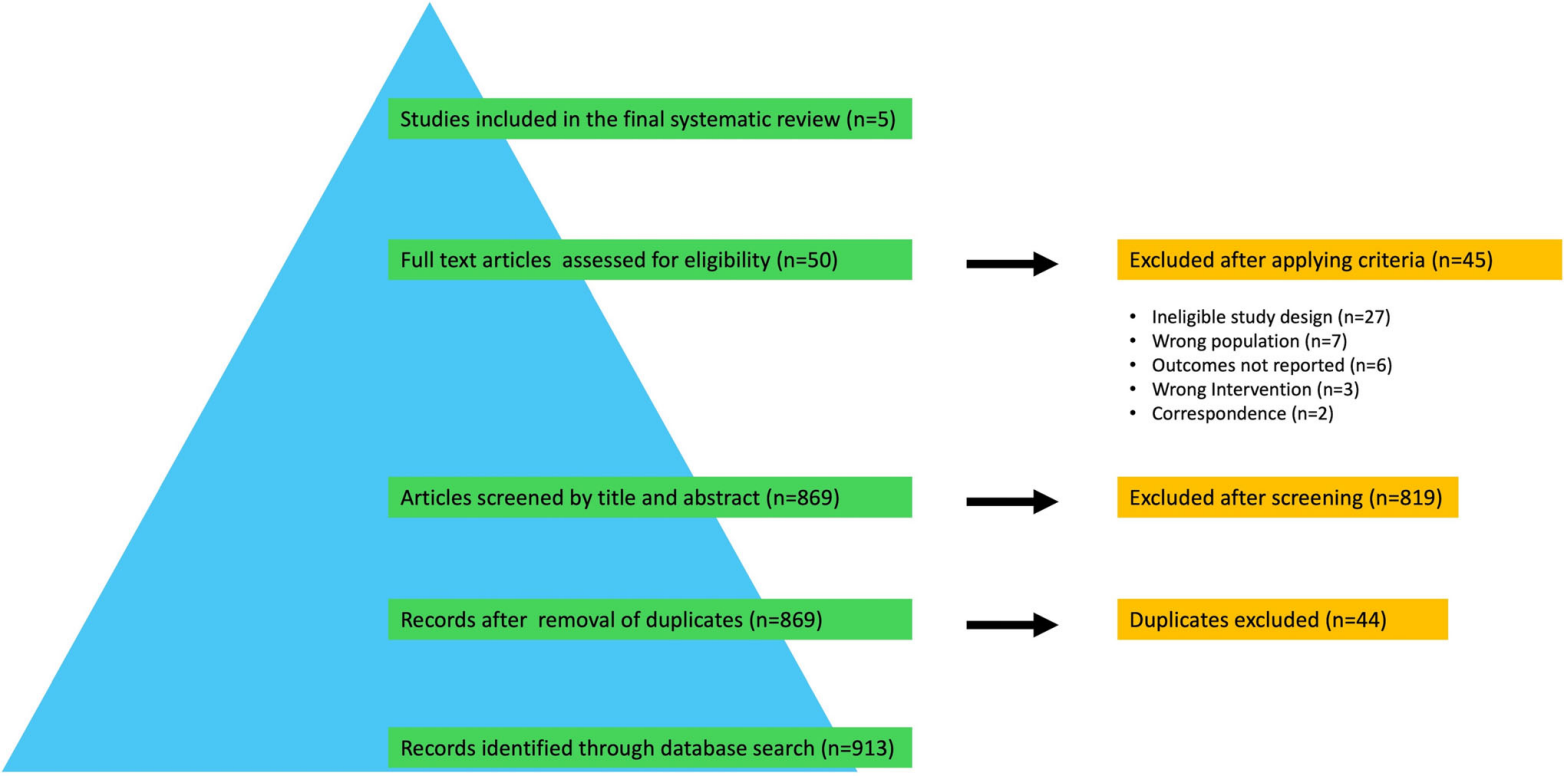
PRISMA-based flow diagram demonstrating the study selection process

Titles and abstracts were screened independently by two reviewers, followed by full-text assessment of potentially eligible studies. Inter-rater agreement was quantified using Cohen’s kappa (*κ*) at the title/abstract and full-text stages (*κ* = 0.91 [*N* = 869] and *κ* = 0.77 [*N* = 50], respectively). Disagreements occurred in 11 (1.27%) records at the title/abstract stage and in 3 (6.0%) articles at the full-text stage and were resolved through discussion and consensus using the prespecified eligibility criteria; all final inclusion decisions required agreement from both reviewers. Reasons for full-text exclusions were documented and summarized in the PRISMA flow diagram (Fig. 1).

### Data Extraction

For each study, author, year, country, study design, and cohort demographics were recorded, as well as the interval between primary and revision surgery, indications (e.g., recurrent platysmal bands, submental fullness, SMG ptosis, contour irregularities, cobra-neck deformity), and techniques performed (platysmaplasty, SMAS or deep-plane modification, SMG procedure, digastric intervention, fat excision, skin redraping). When available, adjunctive procedures, follow-up duration, outcome measures, complications, and re-revision were also noted. Percentages were calculated using available denominators due to missing reporting in the primary studies.

### Risk of Bias and Data Synthesis

Most eligible evidence consisted of retrospective case series with heterogeneous populations, techniques, follow-up reporting, and nonstandardized outcomes. Therefore, we did not apply checklist-based risk-of-bias scoring. Instead, we performed a structured, study-level methodological appraisal using prespecified domains applied to each included study: (1) clarity of population/eligibility definition for revision cases, (2) clarity of the revision neck intervention description, (3) adequacy and reporting of follow-up for revision cases, (4) outcome definition and ascertainment (including whether neck-specific outcomes were reported), and (5) completeness of complication reporting. These domains were used to contextualize certainty of evidence and to guide interpretation of the narrative synthesis.

Given the small number of studies and substantial clinical and methodological heterogeneity, we did not perform meta-analysis, pooled effect estimates, or quantitative heterogeneity statistics (e.g., *I*^2^). Findings were synthesized narratively, and results were summarized using study-level counts and proportions when explicitly reported. Subgroup comparisons (studies with ≥ 12 months of follow-up; studies with versus without SMG surgery) were presented qualitatively.

## Results

### Study Selection and Demographics

The database search yielded 913 records; after removal of duplicates, 869 titles and abstracts were screened. Fifty full-text articles were evaluated, and only five studies specifically addressing revision neck lift surgery met the inclusion criteria (Fig. 1, Table 1).

**Table 1:** Characteristics of the included studies evaluating revision neck lift procedures and related complications

Author	Year	Country	Type of the study	Number of patients	Gender	Mean age (range)	Follow-up (years) (range)	Type of intervention (n)	Neck specific complication (n)
Castro & Braga [8]	1992	Brazil	Retrospective	19	18 female, 1 male	NR (31–70)	NR (examples 4–6 months.)	Cervical lipectomy (16), Platysmal intervention (13)	NR
Perkins & Gibson [17]	1993	USA	Retrospective	22	NR	54 (37–77)	≥ 12 months (range 6 mo–1 y illustrated).	LS (20), Corset platysmaplasty (17), Platysmal resection (15), Cervical lipectomy (7)	NR
Mendelson & Tutino [18]	2015	Canada	Retrospective	31	NR	NR (?)	NR (11 days–7 y)	All cases required SMG excision	Total: 4 (13%); MMN (3)*
Narasimhan et al. [9]	2016	USA	Retrospective	101	96 female, 5 male	57.4 (?)	NR (examples 6 mo–3 y)	All cases underwent submental approach, median (corset) + lateral plication & skin redraping	Total: 2; MMN: (1), Hematoma requiring surgical drainage (1)
Hodgkinson [16]	2018	Australia	Retrospective	15	NR	NR (examples 6 mo–2 y)	NR (?)	Exploration of previous suture and resuspension	NR
Total	–	–	–	188	–	–	–	–	6 (3.2%)

*F* Female, *M* Male, *NR* Not reported, *SMG* Submandibular gland, *LS* Liposuction, *MMN* Marginal mandibular nerve

^*^Note: The overall complication rate was 13% for secondary/tertiary cases. Only MMN neurapraxia was analyzed separately by group, while other complications, such as sialocele or hematoma, were not reported

In all studies, revision neck lift procedures were frequently combined with adjunctive facial rejuvenation techniques, including blepharoplasty, liposuction or lipectomy, laser resurfacing, and other complementary interventions

Methodological reporting quality varied across these retrospective case series, particularly regarding whether revision cases were enrolled consecutively, whether follow-up was reported specifically for revision patients, and the use of standardized outcome measures; these limitations were taken into account when assessing the certainty of the evidence.

These five retrospective case series included 188 patients. Among patients with sex reported, most were female (95%). Because sex was not reported in three of five studies, this percentage reflects only patients with available sex data. Only one study reported a mean age for revision cases (57.4 years) and the available age ranges across the series spanned 31–77 years [9]. Follow-up was variably reported and rarely stratified for revision cases; only Perkins and Gibson documented a minimum postoperative follow-up of 12 months for all patients [16]. Aesthetic outcomes were inconsistently reported and were described predominantly qualitatively (surgeon-observed statements), with few studies providing objective measurements or validated patient-reported outcome measures.

- Revision indications

The main indications were recurrent platysmal bands, submental fullness or fat malposition, SMG ptosis, and cervical contour or scar irregularities. Perkins and Gibson described secondary submentoplasty performed 6–18 months after the index surgery for recurrent cervical laxity and inadequate submental contour [16]. Castro and Braga reported 19 planned secondary facelifts with a mean interval of 10.8 years; Hodgkinson performed 15 secondary facelifts for recurrent neck laxity after 4–8 years; and Narasimhan et al. noted that all 101 secondary neck lifts in their series were carried out at least a decade after the primary operation [8, 9, 17].

Mendelson and Tutino similarly reported more frequent SMG reduction in secondary or tertiary facelift patients presenting with progressive cervical laxity [18]. None of the studies provided a separate numerical breakdown of early complication-driven revisions versus delayed secondary rejuvenation procedures.

(b) Primary techniques and patterns of failure

Several series provided information on the index operation. Perkins and Gibson reported primary surgery with medium to long skin flap elevation, SMAS plication (89%) or imbrication (11%), routine posterior platysma tightening, selective anterior platysma resection (31%), and midline platysma plication in only 13% [16]. Secondary submentoplasty then consisted of liposuction or direct submental lipectomy, treatment of recurrent platysmal bands by anterior resection, and midline platysmaplasty. In Castro and Braga’s series most patients had already undergone cervical lipectomy, submental platysma treatment, and SMAS manipulation, yet secondary lifting required repeat lipectomy, additional platysmal work, and further SMAS procedures [8].

Hodgkinson’s cohort, treated with a modified Fogli technique, was revised mainly by identifying and re-suspending a descended platysmal fixation suture to Lore’s fascia, often without reopening the submental incision [17]. In Narasimhan et al., 76% of patients who had undergone closed or lateral-only neck techniques without midline plication developed recurrent platysmal bands, whereas only one of 13 patients with primary midline platysmaplasty had new bands at revision [9].

(c) Surgical techniques in revision cases

Platysmal maneuvers were the most frequently reported revision interventions, but the term “platysmal modification” encompassed several distinct approaches: midline corset platysmaplasty/anterior plication, lateral plication or suspension, selective partial resection, and rarely caudal transection. Perkins and Gibson described anterior resection with midline platysmaplasty; Narasimhan et al. emphasized midline plication in the revision setting; and Hodgkinson reported resuspension of a descended platysmal fixation suture to Lore’s fascia without routinely reopening the submental incision [9, 16, 17]. Castro and Braga reported additional platysmal work but did not specify the subtype in revision cases [8]. Because revision-specific denominators for each platysmal subtype were inconsistently reported, technique-level pooling and quantitative comparison between platysmal methods were not possible and this is highlighted as a major limitation. Cervical lipectomy (9.8%) and liposuction (8.5%) were commonly used to address lipodystrophy, targeting persistent jowling, submental fullness, and contour irregularities. Although information on whether the fat excess was supra- or subplatysmal was limited, direct excision of subplatysmal fat and supraplatysmal liposuction were generally considered effective when tailored to the depth of involvement. A more conservative defatting strategy was recommended in revision cases because fibrosis from previous operations and minimally invasive treatments may obscure natural planes and increase the risk of contour deformities, including cobra neck [1, 5, 9, 11–14, 16].

Despite increasing recognition of the SMG’s role in cervical contour, only one of the five series reported performing SMG surgery (13.2%). Mendelson and Tutino reserved gland reduction for patients with persistent submandibular fullness despite appropriate fat management [18].

**Fig. 2:**
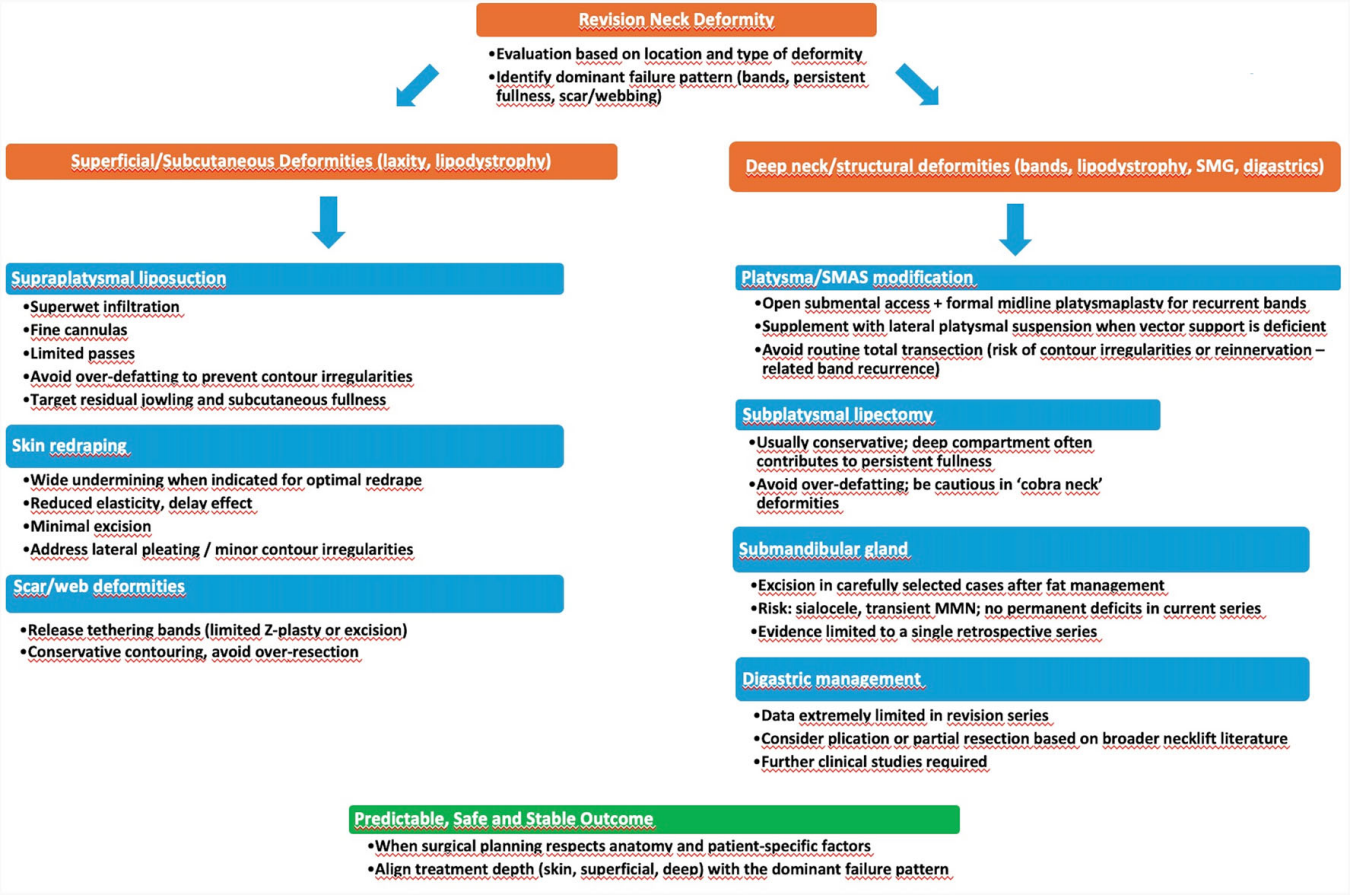
Proposed algorithm for evaluation and surgical planning in revision neck lift surgery

Most revision neck lift procedures were combined with other facial rejuvenation interventions, such as blepharoplasty, brow lift, laser resurfacing, and fat grafting, reflecting a comprehensive cervicofacial approach rather than isolated neck correction. A summary of depth-based evaluation and surgical planning in revision neck lift surgery is illustrated in Fig. 2.

(d) Complications and patient satisfaction

Detailed numerical data on neck-specific complications were available in two series, and a third described early events qualitatively. In Mendelson’s 112 SMG excisions, 31 patients underwent secondary or tertiary face and neck lift; overall complication rates were similar between primary and secondary/tertiary cases (14% vs 13%), with transient marginal mandibular nerve (MMN) neurapraxia in two primary (2.5%) and three secondary/tertiary cases (9.7%), all resolving within 3 months; no lingual or hypoglossal nerve injuries were reported. Early reoperation for hematoma after SMG reduction was required in two patients [18]. Narasimhan et al., reported a 2% complication rate in 101 secondary neck lifts (one transient MMN weakness, one hematoma requiring drainage) [9]. Hodgkinson described two infections and several hematomas without exact numbers [17]. When branch-level information was provided, all facial nerve injuries involved the marginal mandibular branch; none explicitly described cervical branch injury. Across the included series, reported neck-specific complications were mainly transient MMN weakness and isolated hematoma, with no permanent nerve deficits described. Because denominators, follow-up, and complication definitions were inconsistently reported, complication rates were not pooled and are presented descriptively at the study level.

None of the included studies reported objective scales (FACE-Q, VAS, Likert) or structured survey-based outcome measures. Aesthetic results were described only through qualitative, surgeon-observed statements, precluding quantitative analysis of patient satisfaction.

## Discussion

Revision neck lift procedures correct secondary deformities after a primary procedure but are technically more demanding because of anatomical alterations, scar formation, decreased tissue elasticity, and distorted dissection planes [5, 7, 12]. The five available studies, comprising 188 cases, show that the literature consists almost entirely of small, retrospective, and heterogeneous series.

### Patterns of Failure and Principles of Planning

Across the included series, recurrent platysmal banding and persistent submental fullness were the most commonly reported reasons for revision. These deformities typically reflected under-treatment of the platysma in the midline, inadequate lateral suspension, or failure to address subplatysmal lipodystrophy and, in selected cases, SMG prominence at the index operation. Overall, most revision cases appear to represent predictable consequences of incomplete management of the platysma–subplatysmal unit, often compounded by age-related laxity, rather than unexplained late failures.

In practice, planning a revision neck lift begins with identifying the dominant failure pattern and reconstructing the index operation. Careful inspection and palpation should determine whether recurrent banding is due to incomplete midline platysmaplasty, inadequate lateral support, or true subplatysmal laxity. Persistent submental fullness requires distinguishing residual subcutaneous fat from subplatysmal fat and true SMG hypertrophy or ptosis. Skin quality, scar position, and any tethering or webbing influence the extent of undermining and the need for scar revision. Operative reports and photographs from the primary surgery help guide the choice between limited submental access, more extended open approaches.

Patients with recurrent platysmal banding generally benefit from open submental access, extended midline plication or selective partial platysma resection, and lateral platysmal suspension when vector support is deficient. When persistent submental fullness is the main complaint despite a satisfactory skin and SMAS result, attention should shift to the subplatysmal compartment and, in selected cases, to the SMG. Patients whose deformities are dominated by scar bands, webbing, or contour irregularity often require meticulous scar release, limited redraping, and conservative fat contouring rather than aggressive deep-plane maneuvers. In all scenarios, surgeon must balance the desire for maximal correction against the increased risk of nerve injury and wound-healing problems in a previously operated neck.

### Risk Factors and Impact of Minimal Invasive Treatments

Revision neck lift is usually required for recurrent deformity or incomplete primary correction and often occurs 7–11 years after the index procedure as aging changes accumulate [1, 4, 9].

From a technical perspective, inadequate platysma plication or insufficient management of deep neck structures increases the likelihood of recurrence over time [1, 7, 9]. Pelle-Ceravolo highlighted a constant anastomotic network between the cervical plexus and facial nerve branches within the platysma as a potential neuroanatomical factor in recurrent banding [19].

Minimally invasive procedures such as energy-based devices, thread lifting, and fillers may obliterate natural dissection planes and induce fibrosis, complicating dissection. Skouras et al. reported that such treatments increased the difficulty of revision surgery, whereas O’Daniel and Patton noted frequent supraplatysmal fat loss and dense fibrosis [13, 14]. However, none of the revision neck-lift series included in this review systematically quantified the prevalence or impact of prior energy-based or thread procedures. These observations therefore derive from the broader secondary facelift literature and should be interpreted with caution.

Lateral skin pleating and contour irregularities due to insufficient skin excision are also common in secondary procedures and are reported more frequently in patients who previously underwent MACS-lift or other limited-dissection facelifts, in whom wider undermining and redraping may be required [20]. Taken together, the etiology of revision neck deformities is multifactorial.

### Analysis of Surgical Techniques

The surgical strategy in revision neck lift is largely determined by the dominant deformity and the extent of the index operation. Techniques are often grouped into platysmal modifications, fat management, and SMG surgery, but in practice the critical distinction lies in the depth of correction and how these maneuvers are combined.

Platysmal modification is the cornerstone of most revision procedures. Narasimhan et al. showed significantly higher recurrence in patients who did not undergo medial plication at primary surgery, supporting inadequate platysmaplasty as a key driver of secondary deformities [9]. Even with complete platysma transection, bands may recur, likely due to neuromuscular reinnervation [19]. Contemporary practice therefore favors combined medial and lateral plication rather than isolated vector repair, with the understanding that SMAS fibrosis, thinning, or thickening after previous plication may complicate dissection and necessitate individualized planning [7, 12, 21].

Fat management in revision cases should be deliberately conservative, as fibrosis and scar tissue can obscure anatomic planes. Mendelson emphasized that aggressive defatting in secondary surgery can result in contour irregularities and “cobra-neck” deformity, and advocated limited, carefully targeted subplatysmal lipectomy [1, 18]. None of the included revision neck-lift studies, however, provided a specific strategy for correcting cobra-neck deformities.

SMG surgery was reserved for selected patients. In Mendelson’s series, SMG reduction yielded acceptable contour improvement with low rates of sialocele (4.5%) and transient MMN weakness (4.5%), without permanent sequelae, but slightly increased the overall rate of minor complications [18]. Other authors have noted that SMG ptosis becomes more pronounced in secondary deformities and that well-indicated gland reduction can substantially improve the cervicomental profile [22–24]. These data support SMG reduction as a useful adjunct rather than a routine step in revision neck lift. Because SMG procedures were described in detail in only one retrospective series, SMG-related findings should be considered hypothesis-generating rather than practice-guiding. In keeping with a comprehensive cervicofacial approach, revision procedures were frequently combined with other rejuvenation interventions such as blepharoplasty, brow lift, laser resurfacing, and fat grafting.

From a mechanical perspective, the type and extent of the primary technique strongly influence both the pattern of failure and the revision strategy. The series by Perkins and Gibson, Castro and Braga, and Narasimhan et al. suggest that closed or limited lateral neck techniques, liberal liposuction without open submental access, and omission of midline platysmaplasty are associated with higher rates of recurrent laxity, platysmal banding, and submental contour irregularities [8, 9, 16]. In contrast, approaches incorporating open submental access, midline platysmaplasty, and controlled direct fat excision appear to yield more durable midline contour, with fewer patients requiring secondary correction [9]. Accordingly, revision algorithms should prioritize generous submental exposure, formal midline platysmaplasty, and targeted subplatysmal management in patients whose primary procedures were limited to liposuction or lateral tightening, whereas a more conservative, refinement-focused approach may be appropriate for patients previously treated with comprehensive open techniques. With respect to skin management, most authors emphasized that limited additional excision and careful redraping were usually sufficient [1, 7, 9, 25].

### Complication Profile

Across the available studies the most frequent adverse events were transient MMN weakness, sialocele, and hematoma, occurring predominantly in patients who underwent SMG excision and resolving without permanent deficit [9, 17, 18]. Hematoma was often attributed to reopening of emissary veins in the submental region [17]. Although Mendelson’s series reported a higher rate of neurapraxia in secondary cases, Narasimhan et al. found complication rates similar to primary surgery, and all neurological deficits in both series were temporary [9, 18]. Larger facelift series likewise report facial nerve injury rates between 0% and 5.9%, without a clear statistical increase in secondary cases [21].

In the present review, most complications occurred in the cohort that included SMG surgery; the reported rate in that series (approximately 13%) was higher than in those without gland manipulation (around 2%), suggesting an association between SMG procedures and minor, transient complications. With meticulous dissection, limited but adequate exposure, and careful patient selection, revision neck lift can therefore be considered to have a safety profile similar to that of primary surgery [1, 7, 9]. Branch-specific reporting of facial nerve injury was poor, limiting precise comparison of the relative risks to the marginal mandibular versus cervical branches.

### Follow-up Duration and Patient Satisfaction

Follow-up duration was inconsistently reported and rarely stratified by revision status. Only one series documented a minimum postoperative follow-up of ≥ 12 months for all patients [16]. Therefore, the available evidence does not allow robust conclusions regarding long-term durability after revision neck lift. In contrast, objective longevity data from primary facelift cohorts using standardized photographs and validated scoring systems suggest differential regional aging, with partial relapse of neck correction over 5.5 years [26]. Within the reported follow-up intervals, authors generally described outcomes as stable and satisfactory, but assessments were qualitative and nonstandardized, and objective scales or patient-reported measures were largely absent [1, 4, 9].

### Limitations

The findings of this review should be interpreted with caution. Of the 913 records initially screened, only five retrospective case series met the inclusion criteria and specifically reported on revision neck lift. Heterogeneity in patient selection, surgical techniques, follow-up, and outcome reporting precluded formal meta-analysis and limited statistical strength. Complication definitions and revision-specific denominators were inconsistently reported, limiting quantitative synthesis of adverse events. Moreover, aesthetic outcomes were largely described qualitatively, with few objective measurements or validated patient-reported instruments, limiting cross-study comparability and certainty regarding magnitude or durability of improvement. Only one series systematically detailed SMG reduction, so conclusions regarding its indications or risk profile should be considered hypothesis-generating. Nonetheless, by collating and critically analyzing the available data, this review outlines the principal evidence gaps and provides a methodological framework for future research in revision neck lift surgery.

## Conclusion

Revision neck lift may be a safe procedure, with low neck-specific complication rates reported in the available retrospective literature. However, the evidence base is limited to small, heterogeneous case series with inconsistent reporting of technique subtypes, revision-specific follow-up, and most importantly, aesthetic outcomes, which were largely qualitative and surgeon-reported rather than measured with standardized objective or patient-reported instruments. Accordingly, the magnitude and durability of contour improvement, and the comparative effectiveness of specific revision maneuvers, remain uncertain. This systematic review consolidates the fragmented literature on revision neck lift, summarizes current clinical indications and technical trends, and underscores the need for prospective studies using standardized photography, objective measurements, and validated patient-reported outcome measures to better define effectiveness.

## Declarations

### Prospero Registration

This systematic review was prospectively registered in PROSPERO (CRD420251110148).
